# The Effect of Autologous Activated Platelet Rich Plasma (AA-PRP) Injection on Pattern Hair Loss: Clinical and Histomorphometric Evaluation

**DOI:** 10.1155/2014/760709

**Published:** 2014-05-06

**Authors:** V. Cervelli, S. Garcovich, A. Bielli, G. Cervelli, B. C. Curcio, M. G. Scioli, A. Orlandi, P. Gentile

**Affiliations:** ^1^Plastic and Reconstructive Surgery Department, University of Rome Tor Vergata, Via Montpellier, No. 1, 00173 Rome, Italy; ^2^Institute of Dermatology, Catholic University of the Sacred Heart, Rome, Italy; ^3^Institute of Anatomic Pathology, University of Rome Tor Vergata, Via Montpellier, No. 1, 00173 Rome, Italy; ^4^Science Education Department, University of Rome Tor Vergata, Via Montpellier, No. 1, 00173 Rome, Italy; ^5^San Salvatore in Lauro Place, No. 15, 00186 Rome, Italy

## Abstract

To investigate the safety and clinical efficacy of AA-PRP injections for pattern hair loss. AA-PRP, prepared from a small volume of blood, was injected on half of the selected patients' scalps with pattern hair loss. The other half was treated with placebo. Three treatments were given for each patient, with intervals of 1 month. The endpoints were hair re-growth, hair dystrophy as measured by dermoscopy, burning or itching sensation, and cell proliferation as measured by Ki-67 evaluation. At the end of the 3 cycles of treatment, the patients presented clinical improvement in the mean number of hairs, with a mean increase of 18.0 hairs in the target area, and a mean increase in total hair density of 27.7 ( number of hairs/cm^2^) compared with baseline values. Microscopic evaluation showed the increase of epidermis thickness and of the number of hair follicles two weeks after the last AA-PRP treatment compared to baseline value (*P* < 0.05). We also observed an increase of Ki67^+^ keratinocytes of epidermis and of hair follicular bulge cells and a slight increase of small blood vessels around hair follicles in the treated skin compared to baseline (*P* < 0.05).

## 1. Introduction 

Proponents of platelet-rich plasma (PRP) technology suggest that its benefits include an increase in hard- and soft-tissue wound healing. In addition, the role of PRP for the treatment of pattern hair loss has been demonstrated in recent reports [1–4]. In particular, Rinaldi described the use of PRP in alopecia areata (AA). This pilot study suggests that PRP may serve as a safe and effective treatment option in AA and calls for more extensive controlled studies with this method [4]. Uebel et al. showed that pretreatment of follicular units with PRP before transplantation resulted in improved hair growth and density [3]. Activated autologous PRP has been reported to induce the proliferation of dermal papilla cells by upregulating fibroblast growth factor 7 (FGF-7) and b-catenin as well as extracellular signal-related kinase (ERK) and Akt signalling [2]. Anagen-associated angiogenesis has been suggested as one of the important factors in active hair growth [5], due to the secretion of vascular endothelial growth factor (VEGF) by the keratinocytes of the outer root sheath and fibroblasts of the dermal papilla [5–7]. Increased secretion of VEGF influences growth of normal and pathological dermal structures [8]. Tobin et al. reported that the hair follicle mesenchyme exhibits significant hair cycle-associated plasticity. Modulation of these cell interchanges is likely to be important during clinically important hair follicle transformations, for example, vellus-to-terminal and terminal-to-vellus transformations during androgenetic alopecia [9]. Injection of PRP has been demonstrated to improve cutaneous ischemic conditions and to increase vascular structures around hair follicles [1, 10]. Many of the current treatment modalities for pattern hair loss have been shown to modulate angiogenesis and enhance blood flow [11]. The aim is to evaluate the effects of AA-PRP obtained from a small volume of blood on active hair growth. The data we reported proves the clinical efficacy of the treatment with AA-PRP; moreover, patients' satisfaction further confirms the quality of the results. After studying this paper, the reader should be able to (1) prepare AA-PRP, (2) apply PRP intraoperatively, (3) evaluate the clinical effect of AA-PRP on hair growth, and (4) evaluate the histomorphometric effect of AA-PRP on the proliferation of dermal papilla cells.

## 2. Material and Methods

### 2.1. Patients

A total of 10 male patients (age range: 22–60) with male pattern hair loss (MPHL) were treated. The patient characteristics are summarized in Table 1. Patients, who had received topical (such as minoxidil, prostaglandin, analogues, retinoids, and corticosteroid) or systemic treatments for MPHL (such as finasteride, dutasteride, and antiandrogens) in the previous 12 months were excluded. Patients with a propensity for keloids and patients who were immunosuppressed were also excluded. In addition, the numbers of platelets in PRP obtained from all participants were microscopically counted. This was a randomized, TrichoScan evaluator blinded, placebo half-head group study.

The diagnosis of MPHL was established on the basis of clinical and trichoscopic features (more than 20% variability in hair diameter between affected and uninvolved areas), while the extent and stage of MPHL were assessed according to the Norwood-Hamilton classification (as shown in Table 1).

All patients provided written informed consent before participating in the study, which was performed according to the Declaration of Helsinki.

### 2.2. Treatment Protocol

**Figure 1:**
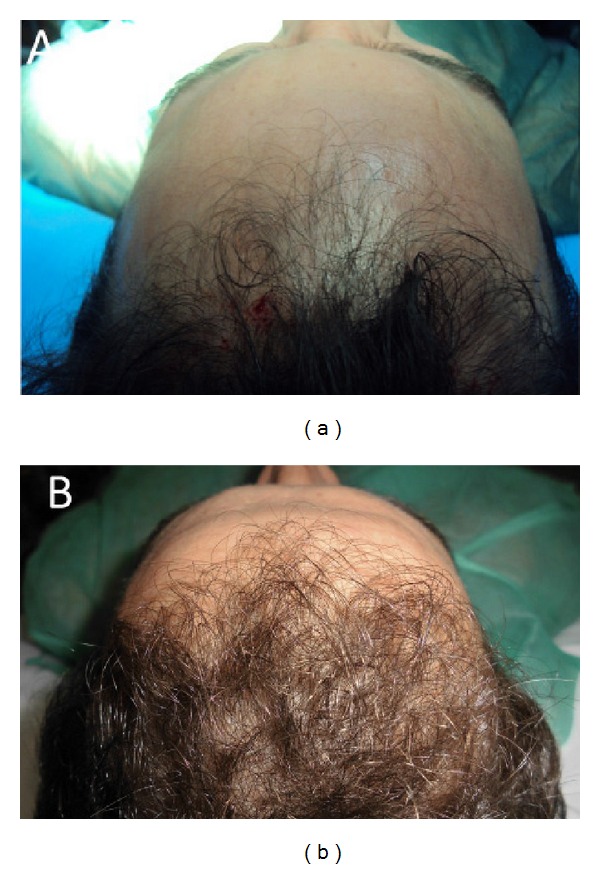
A smoker 34-year-old male patient affected by hair loss. (a): preoperative situation of the frontal line. (b): postoperative situation of the frontal line after two weeks from the last treatment with increase of the hair count and hair density.

**Figure 2:**
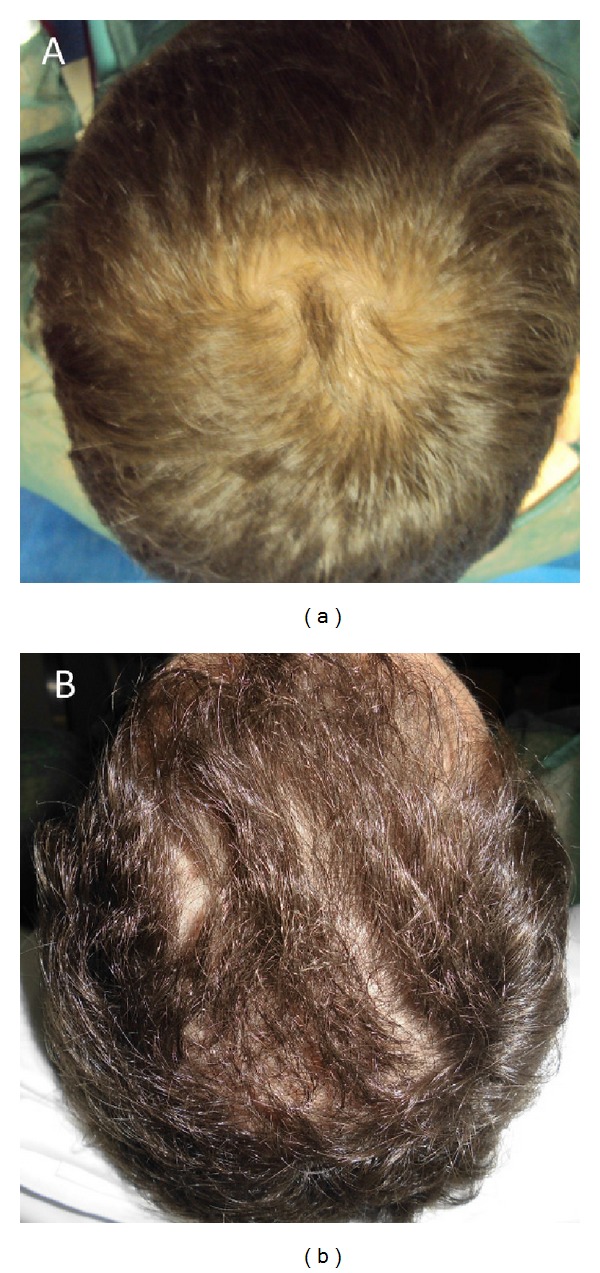
(a): preoperative situation of the scalp. (b): postoperative situation of the scalp two weeks from the last treatment. The picture shows a postoperative situation with increase of the hair count and hair density.

**Figure 3:**
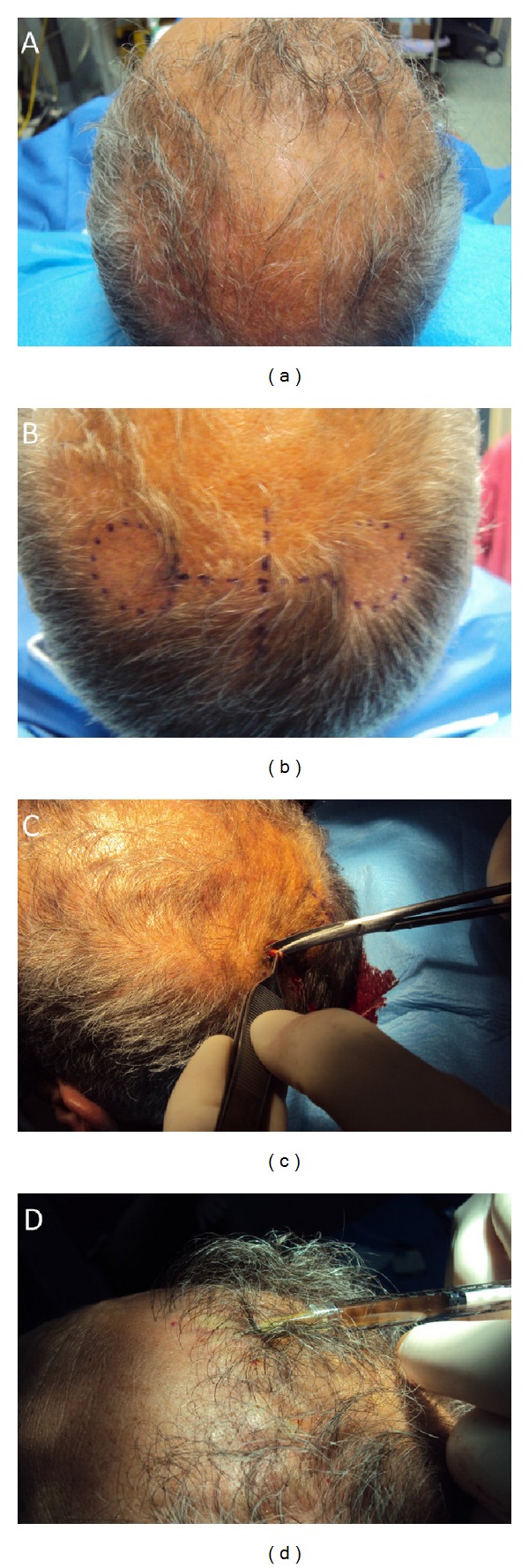
A nonsmoker 52-year-old male patient affected by hair loss. (a): preoperative situation of the scalp with hair loss localized to the temporal and nuchal areas. (b): intraoperative injection with the AA-PRP at 0.1 mL/cm2. (c): intraoperative incisional punch biopsies (3 mm in diameter) of the hair skin fixed in buffered formalin. (d): intraoperative study design.

**Figure 4:**
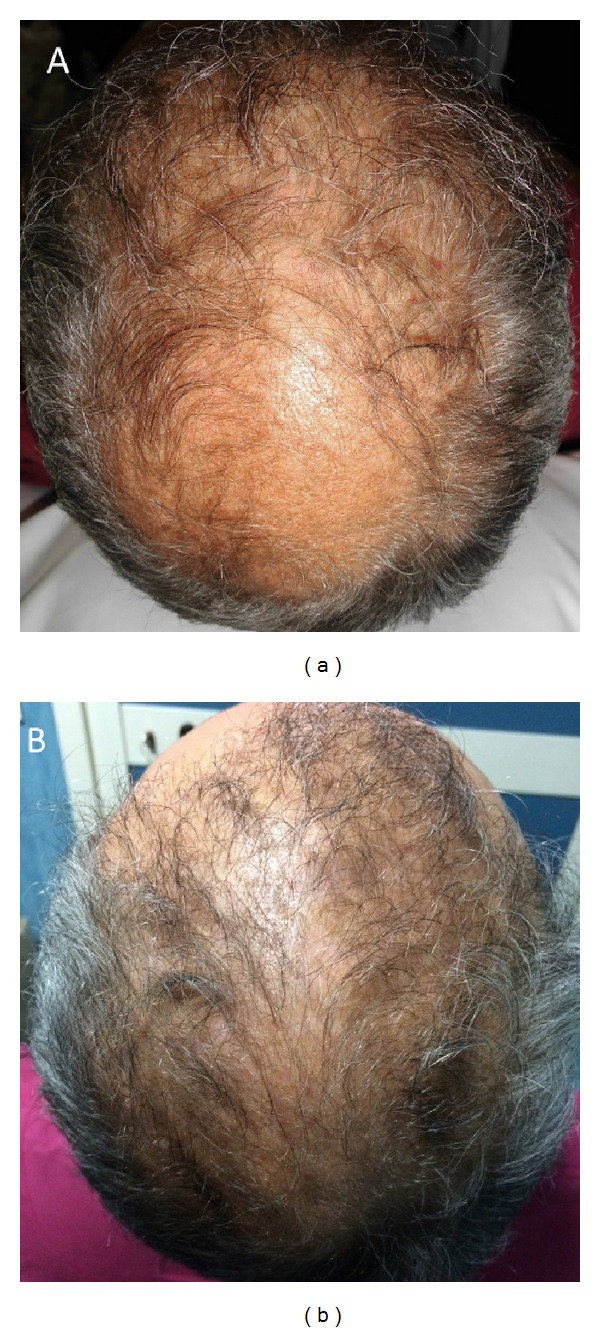
A nonsmoker 52-year-old male patient affected by hair loss. (a): preoperative situation of the scalp with hair loss localized to the parietal and vertex areas. (b): postoperative situation of the scalp two weeks from the last treatment with increase of the hair count and hair density.

AA-PRP was prepared from a small volume of blood (18 cc) according to the method of Cascade-Selphyl-Esforax system, with modifications [12–14]. Briefly, to prepare PRP, blood was taken from a peripheral vein using sodium citrate as an anticoagulant. The current systems for preparing platelet concentrations use various centrifuges (however in this case we used 1100 g for 10 min). AA-PRP was prepared in all cases with approval of the Transfusional Service. Although the method of preparation was not selective and may include leukocytes, the final aim is to obtain a platelet pellet. Growth factors are only secreted once platelet activation begins, which in turn is stimulated by Ca^2+^. To optimize the secretion process, the optimum concentration of Ca^2+^ was previously determined [12, 13]. Then, autologous-PRP not activated (A-PRP) obtained after centrifugation (9 mL) was switched into 10-mL tubes containing Ca^2+^ extracted by Cascade-Selphyl-Esforax Kit. The patients' scalp affected by hair loss was divided in four halves (Figure 8(a)) and cleansed with 70% alcohol, but local anaesthesia was not injected on the treated areas. The AA-PRP was injected on selected areas of the scalp at the amount of 0.1 mL/cm^2^ (Figure 3(d)). AA-PRP injections were injected with the AAPRP only on the frontal areas (Figures 1(b), 9(b), and 10(b)); the parietal area was treated with placebo (Figure 8(b)). The scalps of patients affected by hair loss were divided, respectively, into four parts: frontal, parietal, vertex, and occipital parts. Patients with hair loss localized to the frontal and parietal areas (Figures 1(a), 2(a), 9(a), and 10(a)) were injected with the AA-PRP only on the frontal areas (Figure 1(b)); the parietal area was treated with placebo based on the injection of physiological solution. Patients with hair loss in the parietal and vertex parts (Figures 3(a) and 4(a)) were injected with the AA-PRP only in the parietal part of the scalp (Figure 4(b)); the vertex area was treated with placebo based on the injection of physiological solution. In detail the authors repeat the same numbers of injections in the half treated with PRP and in the half treated with placebo. The analysis of the areas of the scalp treated with PRP and placebo was reported in Figures 8(a), 8(b), and 8(c).

### 2.3. Assessment Criteria

All patients were evaluated in four stages: T0, beginning of study; T1 in 14 weeks; T2, 6 months; and T3, 12 months. The effects of the treatment on hair growth were assessed in all patients with the help of global photography, physician's and patient's global assessment scale, and standardized phototrichograms.

Phototrichograms were performed in all patients by a trained evaluator by means of FotoFinder-video-epiluminescence microscopy in combination with the TrichoScan digital image analysis (Figure 7). TrichoScan is a digital software-supported epiluminescence technique for measuring hair count (number of hairs/0.65 cm^2^), hair density (number of hairs/cm^2^), hair diameter, anagen/telogen ratio, and vellus hair/terminal hair ratio. To determine the quality of hair leading to an increased hair density, it is important to differentiate the number of terminal and vellus hairs. In TrichoScan all hairs with a diameter > 40 *μ*m are categorized as terminal hair, and all hairs with lesser diameter are categorized as vellus hair. In all patients, in both the treatment and control half heads, two transitional areas of hair loss were defined and marked with a semipermanent tattoo for the subsequent trichogram. In the target area hairs were clipped and dyed with hair brown color for ten minutes in order to improve the hair contrast for the analytic software. TrichoScan analysis. The evaluator of TrichoScan analysis was blinded regarding the treatment and control areas of the scalp and not involved in administration of treatment.

### 2.4. Histological Evaluation

**Figure 5:**
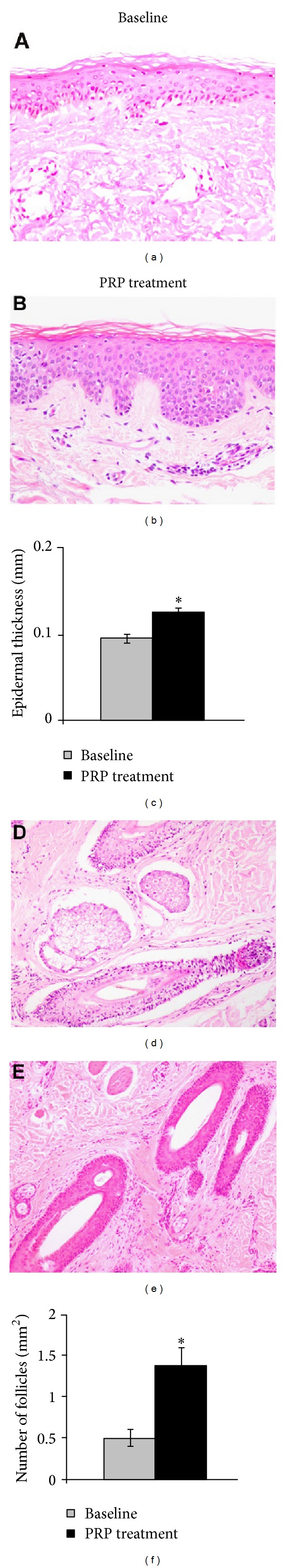
PRP treatment increases the thickness of epidermis and the number of follicles of hair skin. (a) and (b): representative microphotographs of hair skin epidermis at baseline (a) and after PRP treatment (b). (c): bar graph of epidermis thickness. (d) and (e): representative microphotographs of dermal hair follicles at baseline (d) and after PRP treatment (e). (f): bar graph of the number of hair follicles/mm^2^ at baseline and after PRP treatment; ∗ indicates *P* < 0.05. Original magnification: (a) and (b): 200x and (d) and (e): 100x.

**Figure 6:**
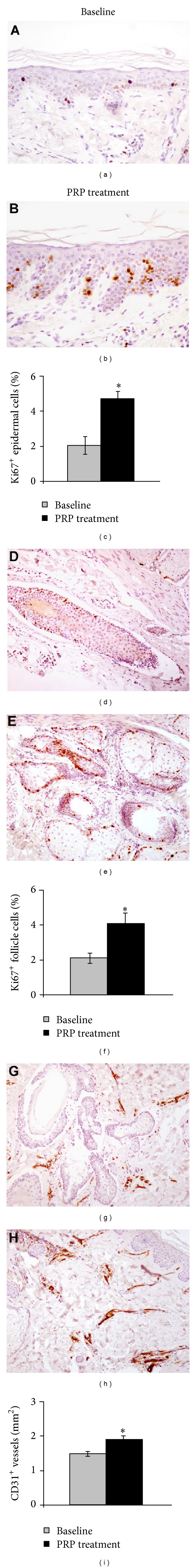
PRP treatment increases proliferation of epidermis basal cells and hair follicular bulge cells. (a) and (b): representative microphotographs of Ki67+ proliferating cells by immunohistochemistry of hair skin epidermis at baseline (a) and after PRP treatment (b). (c): morphometric analysis of Ki67^+^ cells of hair skin epidermis at baseline and after PRP treatment. (d) and (e): representative microphotographs of Ki67^+^ proliferating cells by immunohistochemistry of hair follicles at baseline (d) and after PRP treatment (e). (f): morphometric analysis of the percentage of Ki67^+^ nuclei in hair follicles at baseline and after PRP treatment. (g) and (h): representative microphotographs of CD31^+^ small dermal vessels of hair skin at baseline (g) and after PRP treatment (h). (i): morphometric analysis of CD31+ small dermal vessels of hair skin at baseline and after PRP treatment; ∗ indicates *P* < 0.05. Original magnification: (a) and (b): 200x and (d), (e), (g), and (h): 100x.

**Figure 7:**
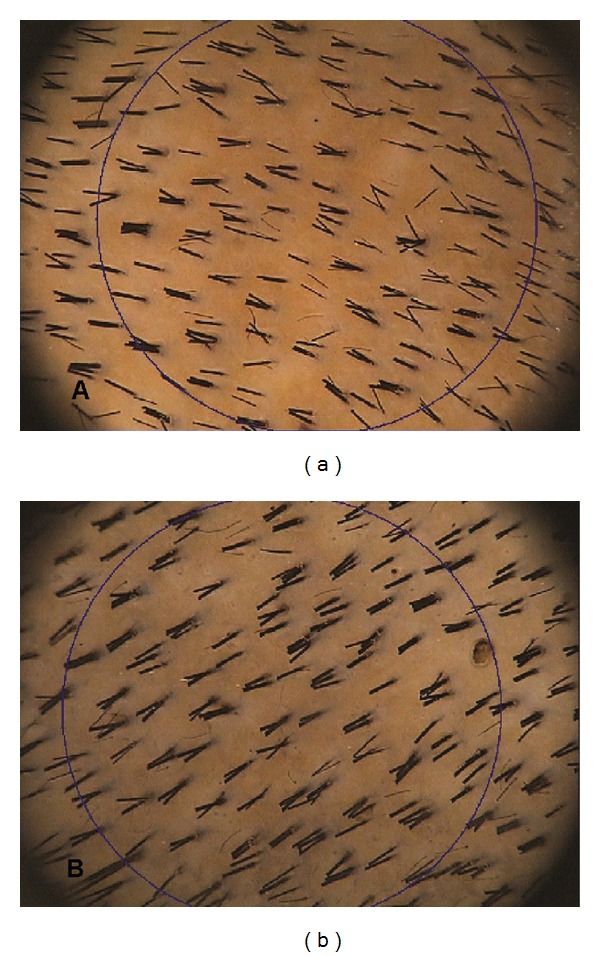
TrichoScan digital image analysis. (a) shows a preoperative hair count 154.5 hairs per cm^2^ and density 237.3 per cm^2^. (b) shows a postoperative hair count 169,0 hairs per cm^2^, and density 259.6 per cm^2^.

**Figure 8:**
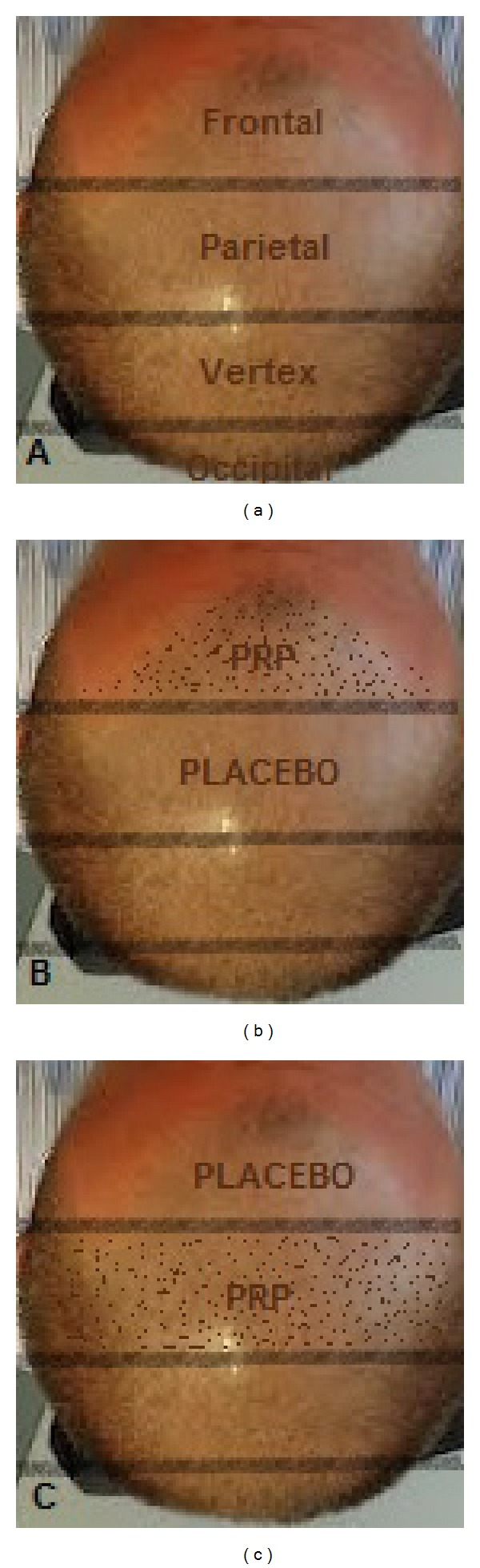
Photos demonstrating the division of the scalp in four halves: frontal, parietal, vertex, and occipital (a). Patients with hair loss localized to the frontal and parietal areas were injected with the AA-PRP only on the frontal areas (b); the parietal area was treated with placebo based on the injection of physiological solution. Patients with hair loss in the parietal and vertex parts were injected with the AA-PRP only in the parietal part of the scalp (c); the vertex area was treated with placebo based on the injection of physiological solution.

**Figure 9:**
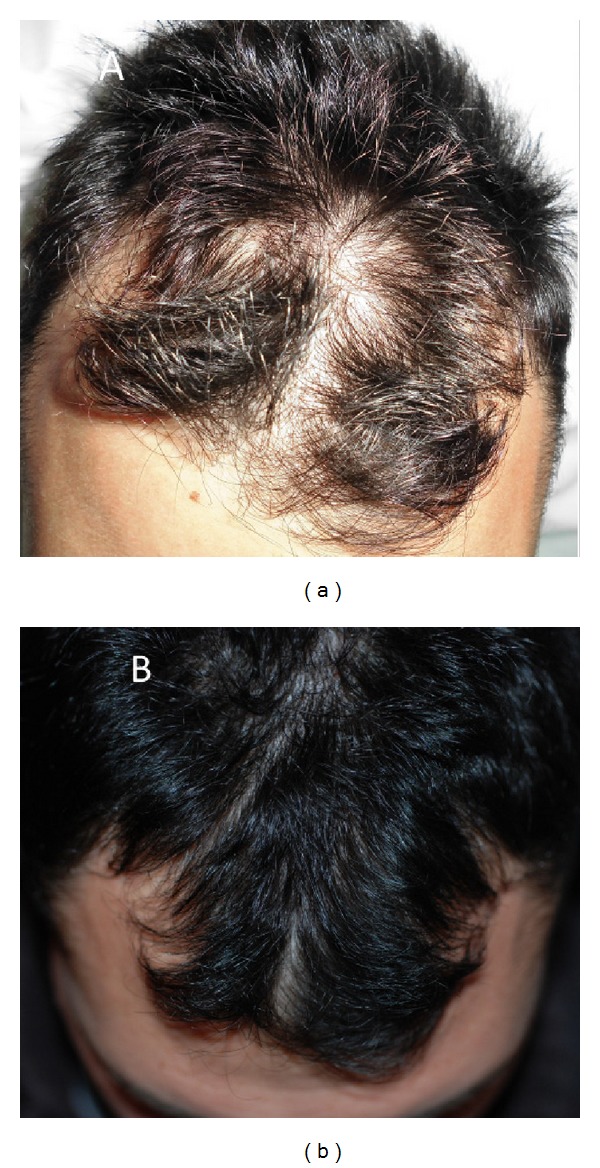
A nonsmoker 52-year-old male patient affected by hair loss. (a) Preoperative situation of the scalp with hair loss localized to the parietal and frontal areas. (b) Postoperative situation of the scalp two weeks from the last treatment with increase of the hair count and hair density.

**Figure 10:**
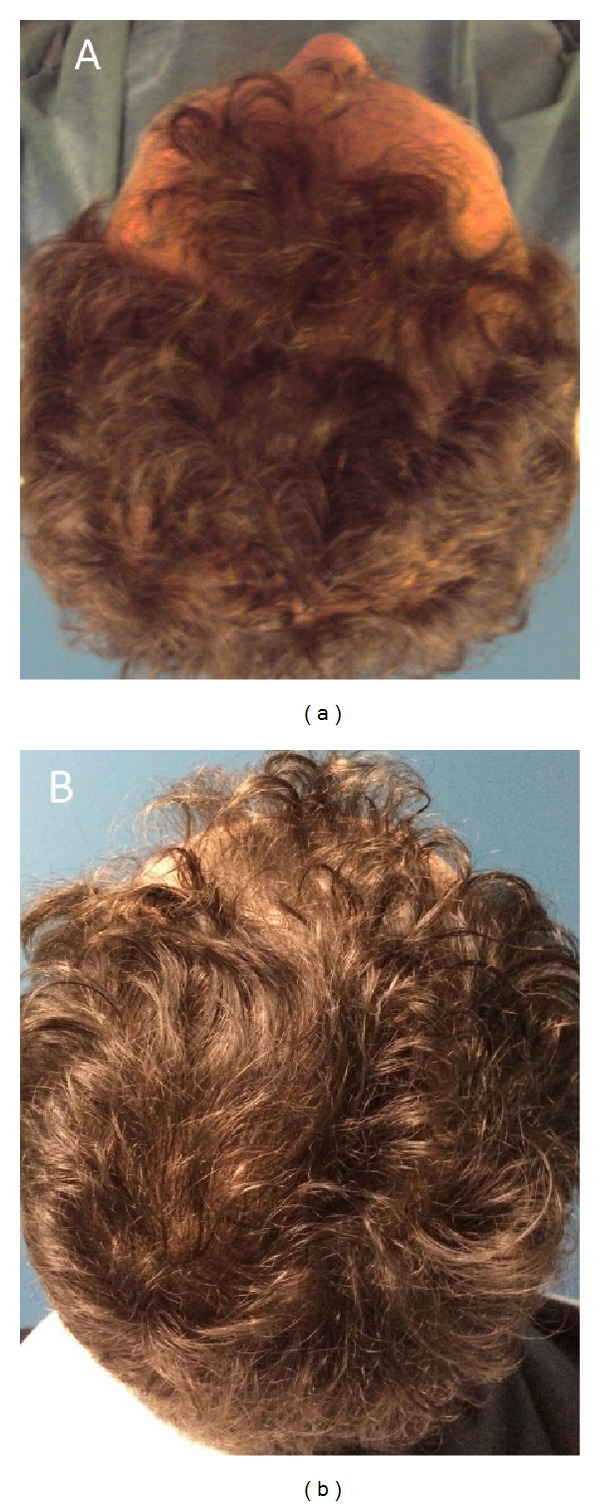
A smoker 42-year-old male patient affected by hair loss. (a) Preoperative situation of the scalp with hair loss localized to the parietal and frontal areas. (b) Postoperative situation of the scalp two weeks from the last treatment with increase of the hair count and hair density.

Incisional punch biopsies (3 mm in diameter) of the hair skin were obtained (Figure 3(c)) at baseline and after two months from the last AA-PRP treatment and fixed in buffered formalin. Morphometric analysis [15] was performed on Haematoxylin-Eosin-stained paraffin serial sections (Figures 5 and 6) by evaluating the thickness of epidermis and the number of follicles per mm^2^, according to the method [1]. About the orientation of skin biopsies, all samples were cut perpendicularly at the surface and embedded making attention to the correct orientation.

### 2.5. Immunohistochemistry

Immunohistochemistry was performed using mouse monoclonal anti-Ki67 (DakoCytomation, Denmark) and anti-CD31 (DakoCytomation, Denmark), with positive and negative controls [16, 17]. The percentage of Ki67^+^ cells in basal layer of epidermis, in outer root sheath of hair follicles, and the number of vessels per mm^2^ were calculated according to morphometric criteria [17].

## 3. Results

### 3.1. Clinical Evaluation of AA-PRP Injection on Pattern Hair Loss

The various hair growth parameters measured after 3 months of the first treatment were compared with the baseline study before treatment (Figures 1(a), 2(a), 3(a), and 4(a)) and between both treatment and control areas. Mean total hair counts, hair density, and terminal and vellus hair densities for the treatment and control areas are listed in Table 2. At baseline, there were no statistical differences in hair count, hair density, and terminal and anagen hair densities between the treatment and control area of the scalp. The results of this study showed a significant increase in the mean hair count for the treatment area after three months (3 months versus 0 month), with a mean increase of 18.0 hairs in the target area compared to baseline, while the control area showed a mean decrease of 2,0 hairs (control versus treatment; *P* < 0.0001). Accordingly, in the treatment area, a mean increase in total hair density of 27.7 (number of hairs/cm^2^) compared to baseline was observed after 3 months and the control area displayed a mean decrease of 3.0 (number of hairs/cm^2^) in hair density at the same time (control versus treatment; *P* < 0.0001). In addition, terminal hair density improved significantly by 27.0 ± 15.3 (number of hairs/cm^2^) in the treatment area (Figures 1(b), 2(b), and 4(b)) compared to baseline, while decreasing by 2.1 ± 12.4 (number of hairs/cm^2^) in the control area of the scalp (control versus treatment; *P* = 0.0003). There were no statistically significant differences in vellus hair density between the study and the control area after three months.

### 3.2. Histomorphometric Evaluation of AA-PRP Injection on Pattern Hair Loss

Microscopic evaluation showed the increase of epidermis thickness (Figure 5(c); *P* < 0.05) in PRP-treated hair skin (Figure 5(b);  *P* < 0.05) after three months from the AA-PRP treatment compared to baseline value (Figure 5(a)). Two-week PRP treatment (Figure 5(e);  *P* < 0.05) was also accompanied by an increase of the number of follicles (Figure 5(f);  *P* < 0.05) compared to baseline value (Figure 5(d)). To better report the effects of PRP, we investigated the proliferation of epidermal and hair follicular bulge cells (Figures 6(b) and 6(e);  *P* < 0.05). After two weeks from the last treatment, we observed an increase of Ki67^+^ basal keratinocytes of epidermis and of hair follicular bulge cells (Figures 6(c) and 6(f);  *P* < 0.05) compared to baseline (Figures 6(a) and 6(d)). PRP treatment (Figure 6(h);  *P* < 0.05) also associated with a slight increase of small blood vessels around hair follicles in the skin treated (Figure 6(i); *P* < 0.05) compared to baseline (Figure 6(g)).

## 4. Discussion 

Current strategies for the treatment of pattern hair loss are mainly focused on promoting cellular proliferation and differentiation during the hair growth cycle. It has been postulated that minoxidil prolongs anagen and increases hair follicle size through stimulation of potassium channels and prostaglandin endoperoxide synthase-1, which increase level of prostaglandin E2 (PGE2) [11]. Minoxidil promotes the survival of dermal papilla cells by increasing Bcl-2/Bax ratio and by activating ERK and Akt [18]. Oral finasteride also induces the prolongation of anagen hairs, which results in gradual thickening and elongation of the hairs [19]. In addition, finasteride has been shown to reduce the pattern hair loss associated with increased expression of caspases and apoptosis inhibitors and therefore it is ultimately suggested to activate anagen hair growth [20, 21]. Antiapoptotic effects of activated PRP have been suggested as one of the major contributing factors stimulating hair growth [2, 22]. PRP-induced activation of antiapoptotic regulators, such as the Bcl-2 protein and Akt signalling, prolongs the survival of dermal papilla cells during the hair cycle [2, 23]. In addition, the upregulation of FGF-7/b-catenin signalling pathways with PRP treatment is suggested to stimulate hair growth by inducing follicular stem cell differentiation as well as prolonging the anagen phase of the hair growth cycle [2, 24].

Kang et al. [25] reported the clinical efficacy of injection of CD34^+^ cell-containing PRP preparation for pattern hair loss. In this study, at three months after the first treatment, the patients presented clinical improvement in the mean number of hairs, 20.5 ± 17.0%, mean hair thickness, 31.3 ± 30.1%, and mean two-point score, 84.4 ± 51.7%, compared with baseline values. At 6 months, the patients presented clinical improvement in mean hair count, 29.2 ± 17.8%, mean hair thickness, 46.4 ± 37.5%, and mean two-point score, 121.3 ± 66.8%, compared with baseline.

In our study, AA-PRP was prepared from a small volume of blood (18 cc) according to the method of Cascade-Selphyl-Esforax system [12, 13]. The authors suggested that a sufficient number of platelets could be obtained in all patients by using an automated PRP preparation system. Giusti et al. demonstrated that the optimal platelet concentration for the induction of angiogenesis in human endothelial cells was 1,500,000 platelets/*μ*L, whereas excessively high concentrations of platelets were suggested to decrease the angiogenic potential [26]. In this study, a mean 1,484,555.6 platelets/*μ*L in the PRP preparation could effectively stimulate follicular and perifollicular angiogenesis, which is suggested to be one of the major factors in active hair growth [5, 11]. Our data suggest that the injection of AA-PRP preparations has a positive therapeutic effect on male and pattern hair loss without major side effects.

## Supplementary Material


Hair growth parameters (hair count, hair density, terminal hair density, vellus hair density) of each patient assessed by TrichoScan analysis for the treatment and control half-head areas at baseline and after 14 weeks.

## Figures and Tables

**Table 1:** Summary of patients' characteristics.

Case	Age	The Norwood-Hamilton classification stage	Injection site
1	20	IIa	Frontal
2	32	IIa	Frontal
3	42	III	Parietal
4	40	III vertex	Parietal
5	41	IIa	Frontal
6	52	IV	Parietal and vertex
7	25	III vertex	Parietal
8	26	III	Parietal
9	28	III	Frontal
10	21	IIa	Frontal

**Table 2:** Relevant hair growth parameters assessed by TrichoScan analysis for the treatment and control half-head areas at baseline and after 14 weeks (T1).

	Treatment area	Control area
Hair count (mean ± SD)		
Baseline	103.6 ± 30.9	111.3 ± 28.9
T1	121.6 ± 34.1	109.3 ± 28.2
Hair density [1/cm^2^] (mean ± SD)		
Baseline	159.4 ± 47.6	171.2 ± 44.4
T1	187.1 ± 52.5	168.1 ± 43.3
Terminal hair density [1/cm^2^] (mean ± SD)		
Baseline	142.7 ± 41.8	152.7 ± 39.7
T1	169.8 ± 47.0	150.6 ± 41.7
Vellus hair density [1/cm^2^] (mean ± SD)		
Baseline	14.8 ± 9.7	16.9 ± 10.4
T1	15.8 ± 8.5	17.4 ± 13.9
